# Applying Machine Learning to Kinematic and Eye Movement Features of a Movement Imitation Task to Predict Autism Diagnosis

**DOI:** 10.1038/s41598-020-65384-4

**Published:** 2020-05-20

**Authors:** Andrius Vabalas, Emma Gowen, Ellen Poliakoff, Alexander J. Casson

**Affiliations:** 1 The University of Manchester, Department of Electrical and Electronic Engineering, Manchester, United Kingdom; 2 The University of Manchester, School of Biological Sciences, Manchester, United Kingdom

**Keywords:** Machine learning, Human behaviour

## Abstract

Autism is a developmental condition currently identified by experts using observation, interview, and questionnaire techniques and primarily assessing social and communication deficits. Motor function and movement imitation are also altered in autism and can be measured more objectively. In this study, motion and eye tracking data from a movement imitation task were combined with supervised machine learning methods to classify 22 autistic and 22 non-autistic adults. The focus was on a reliable machine learning application. We have used nested validation to develop models and further tested the models with an independent data sample. Feature selection was aimed at selection stability to assure result interpretability. Our models predicted diagnosis with 73% accuracy from kinematic features, 70% accuracy from eye movement features and 78% accuracy from combined features. We further explored features which were most important for predictions to better understand movement imitation differences in autism. Consistent with the behavioural results, most discriminative features were from the experimental condition in which non-autistic individuals tended to successfully imitate unusual movement kinematics while autistic individuals tended to fail. Machine learning results show promise that future work could aid in the diagnosis process by providing quantitative tests to supplement current qualitative ones.

## Introduction

Autism is a group of complex developmental conditions characterised by deficits in social skills, verbal and non-verbal communication, and restrictive, repetitive behaviours. However, precise expression of symptoms can vary considerably and there are no universal biomarkers. It is one of the most prevalent developmental disorders affecting approximately 1% of the population, resulting in ~700,000 individuals living with autism in the UK^1^. Currently, its diagnosis relies on clinical experts using observation, interview, and questionnaire techniques, which depend on interpretative coding. The diagnostic process is complex, long and expensive, and the average waiting time between recognising initial concerns and actual clinical diagnosis is more than 3 years in the UK^2^. Thus, valuable time is lost, because early identification and intervention are associated with better outcomes^3^. Although, the majority of autistic individuals receive diagnosis in childhood, many remained undiagnosed until adulthood or not at all. The diagnostic process for the adult population is complicated, as current diagnostic instruments have only been validated for use with children^4^. With adults, clinicians rarely rely on standardised diagnostic methods making diagnosis less accurate, more subjective and lengthier^5^. Thus researching relevant diagnostic criteria for the adult population is critical and listed as one of top ten priorities by the UK’s leading autism research charity Autistica^4^.

In addition to social and communication deficits, current diagnostic criteria recognize repetitive behaviours and movements as core symptoms of autism^6^. However, a broad range of other motor functions are also implicated in autism. Even in the earliest characterization of the disorder, Kanner^7^ recognized unusual motor behaviours and described affected children as “clumsy”. However, only in the last two decades have motor deficits in autism received more attention and increasingly became recognised as important symptoms. In a recent large meta-analysis of studies investigating gait and balance, arm motor function and movement planning, a large and highly significant overall effect size was found showing gross motor impairments in autistic individuals in all examined domains^8^. Motor function deficits are likely to be a good autism biomarker as they occur in the majority of autistic individuals^9–12^, are present from the first year and persist into adulthood^13,14^ and can be measured more directly and objectively, compared to social or communication deficits.

The ability to imitate or copy movements performed by others is also altered in autism. Imitation is a common every-day behaviour important for learning, social interaction and language skills. Metanalyses of imitation studies show deficits in autistic individuals^15,16^, with consistent findings of a reduced rate of spontaneous imitation^17^ and poorer imitation of non-meaningful actions or imitation of manner and style of actions^18–21^. Building on that, several studies have used tasks requiring participants to imitate hand aiming movements while the style of the movement, such as the speed or size, was manipulated^22–24^. These studies used uniform movements allowing them to calculate precise kinematic measures (e.g. velocity, amplitude) by using motion tracking and to objectively compare the performance of autistic and non-autistic individuals. The results consistently showed that imitation precision of the style of the movement is lower in autistic compared to non-autistic adults and also that imitation is not solely an *automatic* behaviour driven by bottom-up processes, but that top-down attentional processes also play a role^22–27^. For example, group differences in imitation of the style of an action are removed if participants are explicitly asked to attend to the kinematics of the action^24^. As reduced imitation of movement style by autistic individuals is reported consistently it is likely to be more universally present and specific to autism compared to other imitation and movement deficits. This suggests that kinematic data from such imitation tasks could offer good discriminability between autistic and non-autistic groups as well as potential for good machine learning (ML) classification performance.

ML methods are well suited for the investigation of heterogeneous and multifaceted conditions such as autism because ML methods, in contrast, to more frequently used traditional univariate methods, make use of complex interactions between multiple variables and classes. However, ML has only recently become more widely used in clinical fields, including autism research. Most of the studies which used ML for autism prediction have used brain imaging data^28^, but studies from other domains also exist including ones which have used movement data.

The studies which have applied ML methods on kinematic data used various tasks: tracking gameplay with sensors on a tablet screen surface^29^, tracking reach-and-throw a ball movements^30,31^ and tracking a simple movement imitation task^32^. The studies had small sample sizes 20 to 82 (mean 40.5) and achieved high classification accuracy rates of 86.7% to 100%. However, the studies used result validation methods which do not necessarily sufficiently control for fitting random noise in the data^33,34^ and did not test the models with new *unseen* data. The studies also did not assess if classification performance was statistically significantly different from random guessing.

The ideal ML model would approximate only the regularities, but not the noise inherent in the training data and then generalise well when tested with new *unseen* data. However, if the model is not sufficiently validated/tested it is unclear how much of its performance is dependent on the noise fitting or on the regularities in the data. Recent ML study surveys suggest that avoiding fitting noise is particularly important when available sample sizes are small — surveyed studies with smaller sample sizes tended to report higher performance estimates^28,35,36^, while theoretically the opposite should be the case^37–39^. In our recent study^40^, in which we asked participants to perform a very simple and short pointing task, we used nested cross-validation, which fully separates training and validation data and has been shown to provide an “*almost unbiased estimate* [of performance]”^33^, even with small sample sizes^36^. In the study the sample size was small (*N* = 46) and 71% classification accuracy was modest compared to other studies which used kinematic data to predict autism diagnosis.

**Figure 1:**
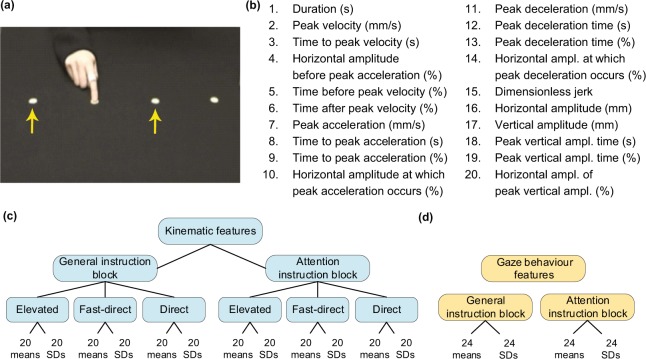
(**a**) Four pointing locations for movement sequences. For kinematic and eye movement data analysis only movements between the two locations indicated with yellow arrows were included. Visual targets are for illustrative purposes only and were not displayed during the video clips or on the table in front of participants. (**b**) Description of kinematic features. (**c**) Kinematic feature structure. (**d**) Eye movement behaviour feature structure. SD - standard deviation. Panel (a) was adapted from^24^, creative commons license CC BY 4.0.

In this study we investigated whether a simple imitation task could discriminate between autistic and non-autistic individuals and characterise autism-specific motor differences. 22 autistic and 22 non-autistic adults performed simple point to point movement sequences after observing them on the screen (Fig. 1a). A motion tracker was employed to collect kinematic data and we also tracked eye movements, while participants observed the movements to imitate. The style of the pointing movements was manipulated, so that movements were performed either in low or high trajectory and either slow or fast. The behavioural results from this experiment, described in detail in Gowen *et al*.^24^, showed that autistic individuals imitated the style of the movement to a lesser extent than non-autistic individuals, consistent with earlier work using this task^22^. Eye tracking also showed reduced visual attention to the movement when it was presented on the screen. These differences, however, diminished when participants were instructed to pay close attention to movement kinematics. Thus, in this study, we predicted that features from the experimental block when participants were instructed to simply copy what they saw would be more discriminative between groups than features from the block when they were asked to pay attention to the movement kinematics.

In contrast to previous ML in autism studies, which have used movement measures, we have also included eye movement behaviour measures, using them separately and in combination. Developing ML models with combined data from different modalities is likely to provide complementary information for predictions and improve classification performance^41^.

A key consideration for the ML work was the reliability of the methods. We aimed to avoid overfitting at the model development stage for the models to reliably predict labels with *unseen* testing data. This is not an easy task with datasets such as ours, which have a large number of measures (features) and a small number of observations (samples). Both, validation to avoid noise fitting^33,35^, and selection of consistent discriminative feature sets used for predictions^42–44^ have to be considered.

To alleviate overfitting in addition to using nested validation we have also tested the models with an independent data. This approach should both provide reliable performance estimates and also show if at model development stage nested validation sufficiently controls noise fitting. The 44-participant sample was split into two parts: data from 30 participants (equally balanced between groups) was used for model development and the remaining data from 14 participants for independent testing of the developed models. As an additional safeguard, we have also assessed if classification performance given by our models was statistically significantly different from random guessing.

To reduce data dimensionality, we have used several traditional feature elimination/selection methods. Those methods are designed to retain features which are most relevant for classification task and some also consider feature redundancy. However, selected feature sets are not always consistent - different feature sets tend to be retained if traditional selection methods are applied on different subsets of the data^42,43^ — especially if the data is high dimensional and the sample size is small^44^. This reduces result interpretability as consistently selected features may aid in understanding and visualisation of the problem. To overcome this issue, we have designed methods aimed at selection stability, with a new “Wrapped *t*-test” method showing good results. This allowed a more meaningful exploration of discriminative features to shed light on autism-specific motor patterns.

In sum we have used a movement imitation task which in previous studies has consistently shown differences between autistic and non-autistic individuals. For ML classification we have combined motion and eye tracking data, and the main aim was reliable ML application. Nested validation was used at model development stage to assure good generalisability when the models were tested with hold-out data. To aid understanding which features were important for classifying individuals as autistic or non-autistic we have used feature selection methods aimed at selection stability.

## Methods

### Experiment and data

We have used separate model development and independent model testing datasets. In the development set, the sample consisted of 15 autistic and 15 non-autistic adults, matched for age, gender, handedness, and IQ. A hold-out testing set, also equivalently matched, consisted of 7 autistic and 7 non-autistic participants (Table 1). The experimental protocol and stimuli are fully described in Gowen *et al*.^24^. During the experiment, all participants were asked to imitate sequences of simple hand movements. Participants first watched then imitated a video shown on a screen while their eye and hand movements were recorded using an eye tracker and a motion tracker. Movement sequences were simple and consisted of two point-to-point movements between three out of four possible locations 15 cm apart on a horizontal straight line, (Fig. 1a). All movements were performed with the dominant hand and only data collected between the locations indicated with arrows in (Fig. 1a) were used. In the videos, vertical amplitude and speed of the movements were manipulated resulting in three conditions: direct, elevated, and direct-fast. Another manipulation was related to attention. Participants first performed a block of imitation trials with a general instruction to simply copy what they saw. Then, in a second block, they were explicitly instructed to attend closely to the kinematic characteristics of the movement (the speed and height). This resulted in six different types of trials: condition(3) × block(2). The experimental procedures involving human subjects described in this paper were carried out in accordance with the Declaration of Helsinki and approved by the University of Manchester research ethics committee, ref: 2017-2541-4204. Informed consent was obtained from all participants.

**Table 1:** Characteristics of training and test samples, *p* - two-sample *t*-test statistic (*α* level 0.05, two-tailed), f - female, m - male, lh - left-handed, rh - right-handed.

	Training sample Autistic	Non-autistic	Autistic vs non-autistic	Independent test sample Autistic)	Non-autistic)	Autistic vs non-autistic	Training vs independent sample
	(n = 15)	(n = 15)		(n = 7	(n = 7		
Age	33.1	32.2	*p* = 0.73	28.16	27.90	*p* = 0.95	*p* = 0.06
IQ	122.0	124.8	*p* = 0.51	122.14	126.29	*p* = 0.46	*p* = 0.82
Gender	3f/12 m	3 f/12 m		4 f/3 m	4 f/3 m		20% f vs 57% f
Handedness	3 lh/12 rh	3 lh/12 rh		1 lh/6 rh	1 lh/6 rh		20% lh vs 14% lh

#### Kinematic features

A Polhemus Fastrak motion tracker was used for kinematic data collection with a single motion sensor attached to the distal phalange of the index finger. The movement was sampled at 120 Hz in X, Y, Z coordinates, filtered with a 120 Hz Butterworth filter, and features based on velocity, acceleration, jerk and amplitude were calculated for each pointing movement, (Fig. 1b). Features were based on the mean and variability (standard deviation (SD)) of each of those measures. In total there were 120 kinematic features per block, 40 per condition (Fig. 1c).

#### Eye movement features

An EyeLink 1000 Plus eye-tracker (SR Research) was used to collect eye movement behaviour data while participants were watching the videos to be imitated. Features were calculated using Data Viewer (SR Research) and MATLAB. The features were based on saccade measures and on visual attention to the finger performing movement sequences (description is given in Supplementary Methods). Both means and variability measures (SDs) for each measure were calculated resulting in 48 features per block (Fig. 1d).

#### Combined features

For combined data, both kinematic and eye movement behaviour features were combined to a single feature set.

### Data normalisation, cleaning

For both datasets, individual trial outliers were removed at the level of participant and group outliers were replaced with group means. Outliers were identified based on the non-recursive procedure recommended by Van Selst and Jolicoeur^45^. In the eye movement dataset, we have also removed trials in which missing data (blinks, pupil/corneal reflection loss) was over 1/3 of the total trial duration. Outlier removal and trials with missing data resulted in 2.3% missing values in kinematic dataset and 7.0% in eye movement dataset. Missing values were replaced with group means. Features were normalised by using standard score (z-score) transformation. Normalisation parameters (means and SDs) were calculated separately from training data to transform hold out data and validation data in each cross-validation (CV) fold during model development.

### Classification algorithm

For classification, Support Vector Machine (SVM) algorithm^46^ was used. It separates the classes by maximising the gap between training examples from each class. The examples in the test data are assigned a label based on which side of the gap they fall. In this study SVM with radial basis function (RBF) kernel was used. Regularisation parameters *C* and *γ* were optimised using grid search with grid parameters set to: *C* = 2^*j*^, where *j* = 1, 2, … 7 and *γ*  = 2^*i*^, where *i* = −1, −2, … −7 and by using 10-fold CV. SVM and grid search were implemented with Libsvm^47^ and Scikit-learn^48^ libraries.

### Validation and performance evaluation

**Figure 2:**
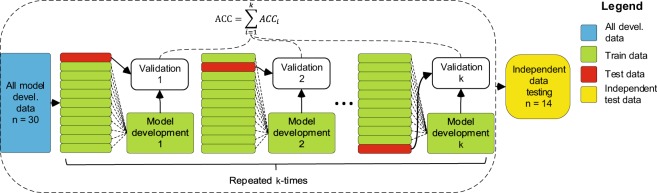
Nested validation for model development and additional testing with independent data. Independent testing (yellow) was applied to each developed model and results averaged. Devel. - development, ACC - overall model accuracy, *ACC*_*i*_. - accuracy in a single CV fold, n - sub-sample size, k - number of CV folds.

At the model development stage, nested cross-validation (CV)^49^ (bounded by the dashed line in Fig. 2) was used for result validation. Nested CV similarly to commonly used K-fold CV^50^ approach validates the results iteratively in CV folds, using all of the available data for training and also reusing all of it for validation. Both validation methods thus are economical and well suited when available data is small as is the case in this study. The nested CV is, however, different from K-fold CV in a significant aspect — it avoids pooling training and validation data. When a nested CV is performed a portion of data is split at the beginning of each CV fold for validation and a model is then developed on the reduced training set, including data normalisation feature selection and parameter tuning. This is repeated iteratively with splitting a different portion of the data for validation, and each time developing a new model for training from scratch until all of the data is used. By using the nested CV approach validation data is separate from model development and in that respect this approach is similar to Train/Test Split testing. Varma and Simon^33^ have demonstrated that nested CV produces almost unbiased performance estimates, while the K-fold CV approach, which pools train and test data, can produce significantly over-optimistic results. In this study 10-fold Nested CV was used and the performance of the model was calculated as a mean performance of ten CV folds. Developed models were tested with independent data (yellow in Fig. 2) by testing each of 10 developed models separately and averaging the results. The process of model development and testing with an independent sample was repeated 50 times to obtain performance distributions, represented by confidence intervals in the graphs, and both model development and testing classification results are reported in the results section.

### Feature selection

In this study, we have used several traditional feature selection methods and developed own methods aimed at feature selection stability. Datasets in which the number of features exceeds the number of observations are problematic for pattern recognition^34,51,52^. However, such datasets are common in neuroimaging, gene expression, behaviour tracking and many other technology-based research areas. In this study both kinematic and eye movement datasets also have more features than samples. Commonly, to overcome this issue, a portion of features are eliminated and in recent years various techniques were developed to accomplish this. Feature elimination/selection achieves several objectives: reliable classifier performance, elimination of irrelevant and redundant features, and selection of stable feature sets.

Using too many features can increase classification error and this effect is exacerbated when sample size is small. Hua *et al*.^53^ performed simulations to find an optimal number of features as a function of sample size for different classifiers and found that with training sample sizes comparable to the ones used in this study an optimal number of features is ≈10 and in this study, with each feature selection method, we reduced feature number to 10.

Feature selection/elimination addresses not only classification robustness but also allows to eliminate features which are redundant or irrelevant for a classification task. Feature elimination methods used in this study can be subdivided into filter and wrapper methods. Filter methods simply apply a mathematical rule which addresses feature relevance, redundancy, or both for feature ranking and highly ranked features are selected for predictions. In wrapper methods, the performance of a classifier is a criterion for feature selection/elimination. This way, feature selection is *wrapped* around a classification model and finds a feature subset giving highest classification performance.

In this study, we have used several traditional filter feature selection methods. These include Student’s *t*-test, which considers only feature relevance by simply ranking features based on how different feature means are between the two classes, as well as two methods which in addition to feature relevance also consider feature redundancy. ReliefF weighs features by considering their interactions^54^. It uses the K-nearest neighbour method to weigh-up features which discriminate best from the neighbours of the different class. mRMR (minimum redundancy maximum relevance) selects features which discriminate categories well but are dissimilar to each other^55^. Both minimum redundancy and maximum relevance criteria are based on mutual information. We have also used a wrapper method SVM-RFE^56^, which eliminates a set number of features which are deemed least important for separating classes by an SVM algorithm, in a number of iterations.

The selected most relevant features may aid in understanding and visualisation of a particular problem and may be useful for biomarker discovery. However, an issue of feature selection stability exists. Frequently, by using different feature selection methods or different subsets of data in a training sample (e.g. in different CV folds), selected features do not match, although classification performance may be comparable^42,43^. A major contributor to feature selection instability is small sample/high dimensional data^44^. To measure feature selection stability we used Kuncheva’s index (KI)^57^. It shows similarity of feature sets as follows:

1 $$KI=\frac{2}{m(m-1)}\mathop{\sum }\limits_{i=1}^{m-1}\,\mathop{\sum }\limits_{j=m+1}^{m}\,\frac{(|{S}_{i}\cap {S}_{j}|l)-{k}^{2}}{k(l-k))},$$

where *m* is a number of feature subsets for similarity calculation, *l* is a number of features in a full dataset, *k* is a number of features in each subset (must be of equal cardinality). KI takes values between −1 and 1, with −1 meaning no overlap between feature subsets and 1 meaning that all feature subsets are identical.

Traditional feature selection methods consider feature relevance and/or redundancy, but selection stability is rarely explicitly considered. Therefore, below we present three approaches which aim to increase feature selection stability.

#### Ensemble feature selection

**Figure 3:**
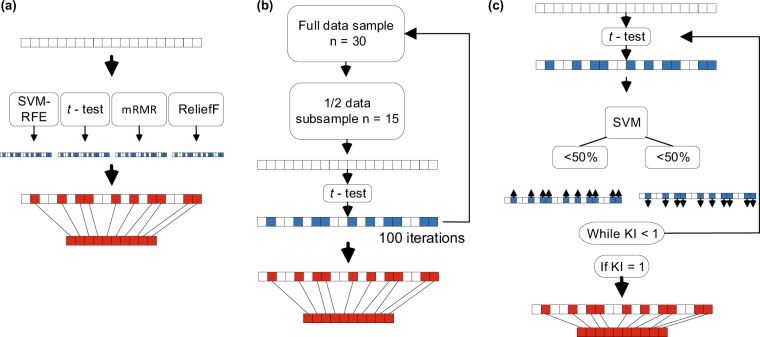
Overview of our three feature selection methods (**a**) Ensemble — rankings of four feature selectors were combined and the 10 highest ranked features were selected. (**b**) *t*-test with bagging — *t*-test feature selection was performed 100 times on random subsamples of size *n*/2 and the 10 most frequently selected features in all the subsamples were selected. (**c**) Wrapped *t*-test — 10 features were selected using *t*-test ranking and their classification performance was assessed with an SVM classifier. If the accuracy was >50%, those features were made to be more likely to be selected in the future iterations by adjusting their *t*-statistics up (or adjusted down if accuracy was <50% making them less likely to be selected). Adjustments were accumulated until Kuncheva’s index (KI) was equal to 1 in 100 consecutive iterations. Blank box — initial feature set; blue/white box — ranked features; red/white box — combined ranks; red box — final feature set.

One way to improve the generalization of classifier predictions is to aggregate the predictions of multiple classifiers^58^. We have applied a similar approach to feature selection by combining different feature selectors. SVM-RFE, *t*-test, mRMR and ReliefF rankings were combined by simple voting and 10 highest ranked features were selected (Fig. 3a).

#### *t*-test with bagging

Meinshausen and Bühlmann^59^ proposed “stability selection” as a technique to improve feature selection stability. A general idea of the technique is that, instead of applying a selection algorithm on a whole dataset to select a feature set, feature selection is performed multiple times on random subsamples of the data. In this study, we have combined this approach with *t*-test ranking. Features were ranked 100 times on the random subsamples of the data of size *n*/2 and the final feature set comprised of 10 most frequently selected features in all of the subsamples (Fig. 3b).

#### Wrapped *t*-test

In this work, we introduce a new method which is also centred on feature selection stability and combines aspects of both wrapper and filter approaches. Instead of removing or adding features based on classification performance, as in traditional wrapper methods, we adjusted ranking statistics of a filter selector by a small magnitude in multiple iterations until a ranking algorithm consistently selected identical feature sets (Fig. 3c). In this study, we have used the absolute value of Student’s *t*-test (two-sample) statistic for ranking. In the first iteration, we have selected 10 features with highest *t*-statistics and then in subsequent iterations, we adjusted *t*-statistics for those features based on classification performance in the outer 10-fold nested CV loop. *t*-statistics were adjusted up or down if classification accuracy was above or below 50% (random guessing level for a balanced two-class data set). Adjustments from all iterations were summed until ranking consistently selected identical feature sets in 100 consecutive iterations. Adjustment magnitude of 0.0001 was chosen because it allowed the algorithm to converge in a manageable number of iterations (<100,000). Although this algorithm is computationally demanding, in the end, it provides a single consistent feature set. It is advantageous compared to other methods as a single consistent set allows a clear interpretation of what measures were important for separating classes.

### Result significance

Statistical result significance was assessed with permutation testing. The labels of the data samples were randomly permutated 100 times and empirical *p*-statistic calculated as in Ojala and Garriga^60^. A significance level of 0.05 was used.

## Results

### Classification performance using data from general instruction and attention instruction experimental conditions

**Figure 4:**
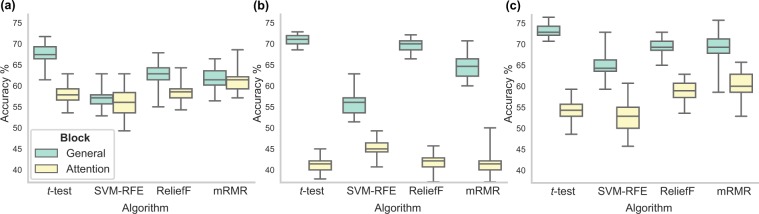
A box and whisker plot showing accuracy distributions of four algorithms with different feature selection using (**a**) kinematic, (**b**) eye movement behaviour, and (**c**) combined data. Green - general instruction block, yellow - attention instruction block.

Classification performance of all feature selection algorithms, followed by the SVM-RBF classifier, in the general instruction block was higher than in the attention instruction block. The difference was moderate for kinematic data (Fig. 4a and Table 2) and more marked for eye movement behaviour (Fig. 4b and Table 3) and combined data (Fig. 4c and Table 4). Based on these results for further analyses we have used only data from the general instruction block.

**Table 2:** Kinematic data classification results. Acc. - accuracy, n.s. - not significant.

Algorithm	General instruction block				Attention instruction block			
	Development		Testing		Development		Testing	
	Acc.	KI	Acc.	*p*	Acc.	KI	Acc.	*p*
*t*-test	66%	0.66	68%	<0.01	66%	0.73	58%	n.s.
SVM-RFE	71%	0.44	57%	n.s.	62%	0.41	56%	n.s.
ReliefF	60%	0.53	63%	n.s.	63%	0.66	58%	n.s.
mRMR	56%	0.21	62%	0.03	66%	0.35	61%	n.s.

**Table 3:** Eye movement behaviour data classification results. Acc. - accuracy, n.s. - not significant.

Algorithm	General instruction block				Attention instruction block			
	Development		Testing		Development		Testing	
	Acc.	KI	Acc.	*p*	Acc.	KI	Acc.	*p*
*t*-test	77%	0.77	71%	0.02	61%	0.63	41%	n.s.
SVM-RFE	65%	0.46	56%	n.s.	59%	0.47	45%	n.s.
ReliefF	76%	0.73	70%	0.02	57%	0.60	42%	n.s.
mRMR	77%	0.36	65%	n.s.	62%	0.25	42%	n.s.

**Table 4:** Combined data classification results. Acc. - accuracy, n.s. - not significant.

Algorithm	General instruction block				Attention instruction block			
	Development		Testing		Development		Testing	
	Acc.	KI	Acc.	*p*	Acc.	KI	Acc.	*p*
*t*-test	75%	0.76	73%	<0.01	65%	0.62	54%	n.s.
SVM-RFE	66%	0.32	65%	0.03	62%	0.34	53%	n.s.
ReliefF	76%	0.72	69%	0.01	63%	0.64	59%	n.s.
mRMR	68%	0.31	69%	0.01	62%	0.33	60%	n.s.

Overall, classification accuracies at the model development stage (columns *Development*) were comparable to accuracies when those models were used to predict labels in independent test data-set (columns *Testing*), Tables 2, 3, and 4. Nested cross-validation was sufficient to control overfitting and produced results which generalised well to the independent test sample.

An algorithm using the simplest *t*-test feature selection outperformed all other algorithms in terms of classification accuracy and feature selection stability (KI) and this was the case for all data types (Fig. 4). In our previous study where we used kinematic features from a simple movement task^40^ we have obtained similar results — *t*-test feature selection outperformed other algorithms. Additionally, in other studies which had high dimensional/small sample datasets *t*-test consistently outperformed other algorithms in terms of selection stability and classification accuracy^61,62^. Taking this into consideration in the next section, we report the results of two variations of *t*-test feature selection: using bagging, combining *t*-test with wrapper selection approach, as well as, an ensemble of feature selectors which includes *t*-test.

### Improved feature selection

**Figure 5:**
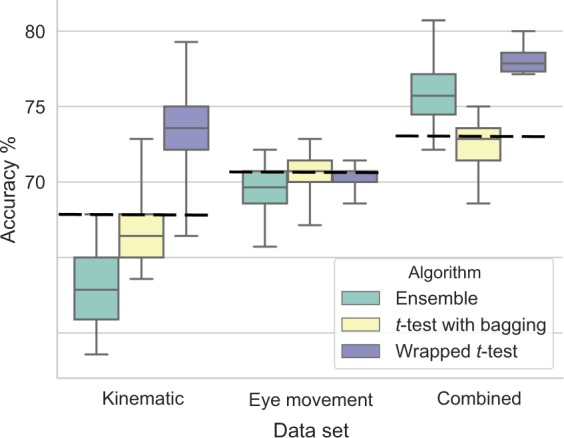
A box and whisker plot showing accuracy distributions of three algorithms with different feature selection using kinematic, eye movement behaviour, and combined data. Dashed lines show classification performance using *t*-test feature selection alone.

The idea behind an ensemble method is that a combination of output produced by multiple algorithms is potentially better than the output of a single algorithm. Ensembles have been shown to produce less variable and more robust results, especially with high dimensional/small sample data^63^. With our datasets, the ensemble method outperformed SVM-RFE, ReliefF, and mRMR, both in classification accuracy and feature selection stability. *t*-test alone, however, produced very similar results to the ensemble (Figs. 4 and 5).

The *t*-test feature selection consistently produced the most stable feature sets which nearly consistently outperformed other algorithms, thus we further explored two variations of *t*-test algorithm, with the aim of improving feature selection stability. First, we used *t*-test with bagging. Instead of applying the *t*-test algorithm on a whole data, feature selection was performed 100 times on the random subsamples of the size *n*/2. The results of *t*-test with bagging, however, did not show improvement on *t*-test alone both in stability or performance (Figs. 4 and 5).

**Figure 6:**
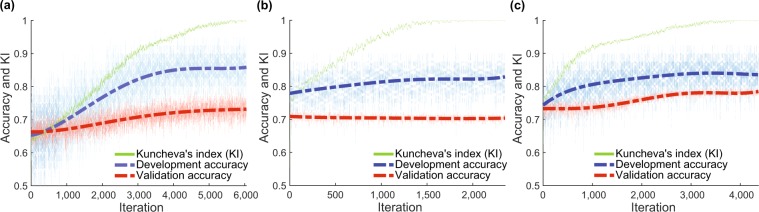
Illustration of Wrapped *t*-test algorithm performance over successive iterations with (**a**) kinematic, (**b**) eye movement behaviour, and (**c**) combined datasets. In each model development iteration feature selection was performed 10 times – in each nested CV fold. Iterations were performed until KI was equal to 1 in 100 subsequent iterations. Thick dash-dot lines show fitted 5^*th*^ order polynomial trend.

Finally, we have used our new “Wrapped *t*-test” method which combines features of both filter and wrapper methods. Figure 6 shows that cumulative adjustments of *t*-statistics progressively led to more stable feature selection demonstrated by increasing KI and also progressively better classification accuracy at a model development stage. Importantly, classification accuracy also increased on the independent dataset with the kinematic data (Fig. 6a) and combined data (Fig. 6c). There was no such effect with eye data (Fig. 6b). With kinematic data classification accuracy was 73%, with a sensitivity of 88%, specificity of 59%, and *p* < 0.01, with eye data 70% accuracy, 43% sensitivity, 97% specificity, *p* = 0.02, with combined data 78% accuracy, 57% sensitivity, 99% specificity, *p* < 0.01. This approach produced the best classification accuracy on kinematic and combined datasets (Table 5). However, it did not show improvement of the eye dataset. This was likely because eye features were more similar and inter-correlated ($\bar{{r}}=0.48$) than kinematic ($\bar{{r}}=0.20$) or combined ($\bar{{r}}=0.20$) features, and the number of features was considerably lower.

**Table 5:** Classification results with ensemble, *t*-test with bagging, and Wrapped *t*-test feature selection.

Algorithm	Kinematic data				Eye movement behaviour data				Combined data			
	Development		Testing		Development		Testing		Development		Testing	
	Acc.	KI	Acc.	*p*	Acc.	KI	Acc.	*p*	Acc.	KI	Acc.	*p*
Ensemble	67%	0.61	63%	0.05	76%	0.71	70%	<0.01	71%	0.59	76%	<0.01
*t*-test with bagging	65%	0.60	67%	0.03	77%	0.74	71%	0.03	74%	0.75	72%	<0.01
Wrapped *t*-test	85%	1.00	73%	<0.01	83%	1.00	70%	0.02	84%	1.00	78%	<0.01

### Discriminatic kinematic features

**Figure 7:**
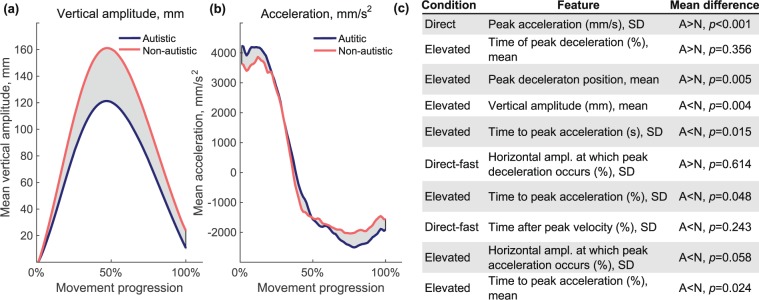
(**a**) Movement vertical amplitude and (**b**) acceleration averaged for autistic and non-autistic participants in the elevated experimental condition, general instruction block. Shaded areas show the difference between groups. (**c**) Features selected with Wrapped *t*-test selection method. Mean difference column shows whether the mean for particular feature was greater for autistic (A) or non-autistic (N) class and gives a *p*-value of two-sample *t*-test.

Here we interpret why selected kinematic features were salient for classification. For similar interpretation of selected discriminative features in eye movement and combined datasets see a Supplementary Note. Wrapped *t*-test feature selection was stable — we repeated feature selection on randomly sub-sampled data ten times and consistent feature sets were selected each time. Among the selected features in the kinematic dataset, 7 out of 10 features were from the elevated condition (Fig. 7c). This corresponds with the behavioural results in previous studies, which used imitation tasks and have shown reduced vertical amplitude modulation in individuals with autism^22–24^. Our data also shows a significant difference in movement vertical amplitude between autistic and non-autistic individuals when they were asked to imitate elevated trials, *t*(42) = 3.0, *p* = 0.004, *d* = 0.93, Fig. 7a. There were also differences in the acceleration profile between autistic and non-autistic individuals in elevated trials (Fig. 7b). Autistic individuals reached peak acceleration earlier in the movement, *t*(42) = 2.5, *p* = 0.017, *d* = 0.75, and peak deceleration later in the movement, *t*(29.4) = 3.0, *p* = 0.006, *d* = 0.90. This corresponds with the selected discriminative feature set as six out of ten features were acceleration/deceleration measures in elevated condition. Overall, both discriminative features and statistical differences suggest that non-autistic individuals reduced acceleration and deceleration and increased vertical amplitude in order to copy unusual elevated movement kinematics, while autistic individuals tended to retain their usual style of movement.

## Discussion

Differences between autistic and non-autistic individuals have been shown across a broad range of movement and movement imitation tasks^8,15,16^. Among the more consistent findings is reduced imitation of movement style/manner by autistic individuals^18–20,22,23^. In this study, we have used an imitation task in which movement style was manipulated and explored whether kinematic and eye movement behaviour measures could predict autism diagnosis. Developed models achieved a classification accuracy of 73% with kinematic data and 70% with eye movement data. We have also combined data from both kinematic and eye movement behaviour modalities. This provided complementary information for predictions and models on combined data gave the highest classification performance of 78%.

To date, only a handful of ML studies used kinematic data for autism prediction. All studies had small sample sizes and achieved high classification accuracy. Models developed with small sample/high dimensional data are prone to fit noise and not necessarily an underlying pattern separating classes^51,64^. However, completely separating training and validation data (treating validation data as *unseen*) is sufficient to control overfitting and produce reliable performance estimates^33,36^. To the best of our knowledge, those studies did not avoid pooling training and validation data while developing their ML models and did not take other steps to control for the fitting of random noise in the data. Classification results were not tested with independent data and researchers did not assess the statistical significance of the results.

Our focus for ML work was the reliability of used methods. We aimed to avoid fitting the noise in the data during model development stage to help assure that the models reliably predict labels with independent/*unseen* data during the testing stage. This was, however, not an easy task because of the characteristics of our datasets, which had a small number of samples and a high number of features. Such datasets are problematic for pattern recognition^34,35,51,52^ and both result validation and consistent feature selection required careful consideration.

For result validation at the model development stage, we used nested CV because it was shown to produce reliable results^33^. Moreover, we have shown that nested CV produces reliable results regardless of sample size^36^. In addition to that, we have further verified that our models were not overfitted, by testing the results with an independent dataset. Classification performance at the model development stage was comparable to independent validation performance, showing that nested validation was sufficient to control overfitting. This was the case even though training and independent testing datasets were significantly different in terms of gender composition. There were 20% of females in model development dataset and 57% in the independent testing dataset (Table 1). There are more males than females with autism diagnosis and recent studies suggest that there may be phenotypic gender differences^65,66^.

Reliable feature selection was also a difficult issue to overcome. Traditional filter and wrapper feature selection methods produced only modest classification results, however, ranking feature sets with *t*-test consistently outperformed other methods in terms of feature selection stability and classification performance. Therefore, we developed two feature selection variations based on *t*-test with the main goal to improve feature selection stability. We used *t*-test with bagging by randomly subsampling data and aggregating feature ranks from multiple iterations. This method, however, did not show improvement on using *t*-test feature selection alone. A new “Wrapped *t*-test” algorithm combined aspects of filter and wrapper approaches. We adjusted *t*-statistics used for feature ranking by a small magnitude in multiple iterations based on classifier performance. We ran this algorithm until *t*-test algorithm consistently ranked identical feature sets. With increasing feature selection stability this algorithm increasingly fitted training data, importantly, it also produced a better performance on independent data as well. In addition to good classification performance, this method provided a stable final set of 10 features which has helped to illustrate movement imitation differences in autism.

In the kinematic feature set, selected using a wrapped *t*-test, seven out of ten features were from the trials where movements were performed in elevated amplitude, and the same condition showed significant differences between autistic and non-autistic groups using parametric statistical tests. After watching videos of elevated movements to imitate, autistic individuals performed movements using a lower vertical amplitude than non-autistic individuals. As a consequence, autistic individuals also reached peak acceleration earlier in the movement and peak deceleration later in the movement. These results suggest that autistic individuals tended to retain their usual style of the movement when the movement to imitate had unusual kinematics. This is consistent with a number of studies which shown that while autistic individuals are more able to imitate goals of the action, they are less proficient at imitating the style or kinematics^18–22,24,67^.

In a previous study^32^, which applied ML methods to the data from a similar imitation experiment with different participants, researchers selected exclusively only variability measures as most discriminative kinematic features, although feature selection was performed in a not fully algorithmic way (several feature selection algorithms were combined with selection decisions by researchers). This was not the case in our study with both means and SDs selected (Fig. 7c). However, in full feature sets, we found that autistic individuals tended both to perform movements with greater variability and to pay visual attention to the observed movement more variably. In the kinematic dataset autistic individuals showed higher variability than non-autistic individuals in 73% of 120 variability features (9% statistically significantly at 0.05 *α* level, two-tailed), in the eye movement behaviour dataset in 90% of 48 variability features (42% statistically significantly at 0.05 *α* level, two-tailed). Increased variability is a common finding in autism, reported for reaching movements^68^, hand aiming movements^69^, sustained force^70^, precision grip^71^, walking^72^ and Saccadic eye movements^73^. Increased variability suggests differences in sensorimotor control and is especially apparent with challenging tasks and those requiring precision^70^.

## Conclusion

In this study, we used a movement imitation task, which based on previous evidence, suggested good discriminability between autistic and non-autistic groups. ML classified autistic and non-autistic individuals with 73% accuracy using kinematic measures and with 70% accuracy using eye movement behaviour measures. Moreover, combining measures from both modalities provided complementary information for predictions and gave a classification accuracy of 78%. We have overcome overfitting and stable feature selection issues by using nested validation and feature selection aimed at selection stability and show that even small-sample studies can achieve statistically significant predictions which generalise to *unseen* data. The results show a promise that future work could aid in diagnostic process, by reliably applying ML methods and possibly combining features from several modalities.

## Supplementary information

Supplementary Information.

## Data Availability

The datasets generated and analysed during the current study are available at The University of Manchester repository: 10.17632/fnt6jtc5np.4.
