# Supplementary material for: Applying Machine Learning to Kinematic and Eye Movement Features of a Movement Imitation Task to Predict Autism Diagnosis
Source: Sci Rep. 2020 May 20;10:8346. doi: 10.1038/s41598-020-65384-4 (PMC7239902; doi:10.1038/s41598-020-65384-4)
Supplement: Supplementary file 1 — Supplementary Information. [file 41598_2020_65384_MOESM1_ESM.pdf]

# Applying Machine Learning to Kinematic and Eye Movement Features of a Movement Imitation Task to Predict Autism Diagnosis

Andrius Vabalas[1*], Emma Gowen[2], Ellen Poliakoff[2], Alexander J. Casson[1]

**1** The University of Manchester, Department of Electrical and Electronic Engineering, Manchester, United Kingdom (email: {andrius.vabalas, alex.casson}@manchester.ac.uk)
**2** The University of Manchester, School of Biological Sciences, Manchester, United Kingdom (email: {emma.gowen, ellen.poliakoff}@manchester.ac.uk)

## **Supplementary Methods.** Description of eye movement behaviour features.

Eye movement behaviour features were based on visual attention to the finger performing movement sequences and were calculated either for the whole movement duration between the two locations investigated (Fig. 1 in the main paper), when the finger was static on the locations, or when the finger was moving between locations. Attention to the finger was measured either as distance from the fingertip, horizontal offset from the fingertip, fixation count to the interest area (IA) around the fingertip, or dwell proportion to the IA. Description of why attention to the fingertip was measured and how IA was defined is fully described in Gowen et al.[1] There were also additional features based on the trade-off between looking to the IA around dynamic finger and looking to the static pointing target locations. Saccade measures based on overall saccade amplitude and velocity were also included. A full breakdown of features is given in Fig. S1.

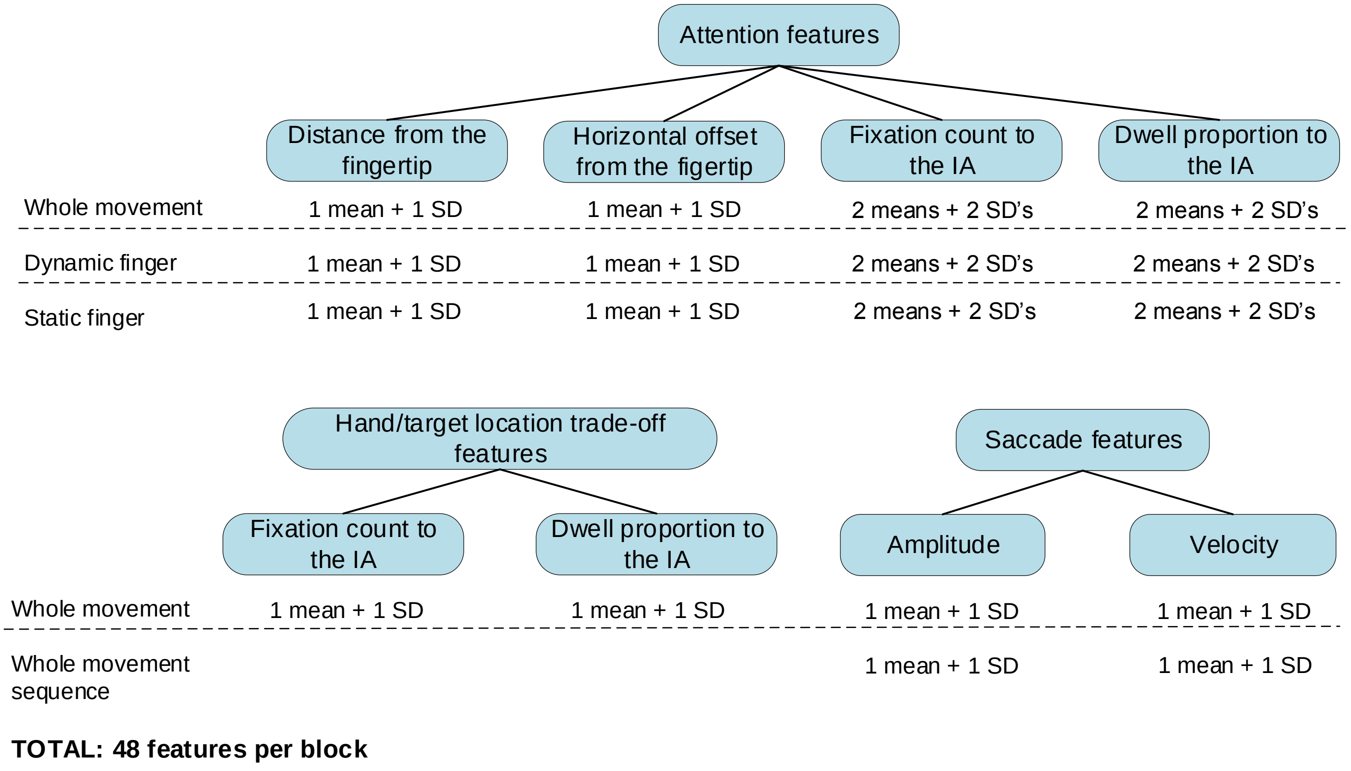


**Figure S1.** Breakdown of eye movement behaviour features. Whole movement — movement between two locations of interest, static finger — part of the movement when the finger was static on the start/end locations, dynamic movement — part of the movement when the finger was moving between the start/end locations, SD — standard deviation, IA —- interest area.

**Supplementary Note.** Discriminative eye movement behaviour and combined features.

Interpretation of why selected features were salient for classification can shed some light on the problem at hand. In the main article text, we gave our interpretation for the selected ten discriminative features from the kinematic feature set. Here we explore the most discriminative features from the eye movement and combined datasets selected using the Wrapped $t$-test method.

### Eye movement dataset

Table S1 shows that all selected eye movement features were based on visual attention to the finger performing movement sequences on the screen for participants to imitate (Fig. 1a in the main text). All ten selected features were similar and between feature inter-correlation of $\bar{r} = 0.70$ was even higher than the inter-correlation in a full 48 feature eye movement dataset ($\bar{r} = 0.48$). All except one feature were variability measures, all showing greater variability in visual attention in autistic compared to non-autistic individuals. The similarity of features did not allow us a detailed analysis of individual features similarly as for kinematic features (see section Discriminative features in the main manuscript). The only inference we could make was that greater variability in visual attention exhibited by autistic individuals was important for classification.

| **EYE MOVEMENT DATASET** | | **COMBINED DATASET** | |
|---|---|---|---|
| **Feature** | **Mean** | **Feature** | **Mean** |
| Whole movement, dwell duration to IA, SD | A>N, $p$<0.001 | Whole movement, dwell duration to IA, SD | A>N, $p$<0.001 |
| Static finger, horizontal offset from fingertip, SD | A>N, $p$=0.003 | Static finger, distance from fingertip, SD | A>N, $p$=0.003 |
| Static finger, dwell duration to IA, SD | A>N, $p$<0.001 | Dynamic finger, dwell duration to IA, SD | A>N, $p$<0.001 |
| Dynamic finger, dwell duration to IA, SD | A>N, $p$<0.001 | Static finger, dwell duration to IA, SD | A>N, $p$<0.001 |
| Static finger, distance from fingertip, SD | A>N, $p$=0.003 | Static finger, horizontal offset from fingertip, SD | A>N, $p$=0.003 |
| Whole movement, distance from fingertip, SD | A>N, $p$=0.006 | Whole movement, distance from fingertip, SD | A>N, $p$=0.006 |
| Whole movement, dwell proportion to IA, SD | A>N, $p$<0.001 | Static finger, dwell proportion to IA, mean | N>A, $p$<0.001 |
| Static finger, dwell proportion to IA, mean | N>A, $p$<0.001 | Dynamic finger, horizontal offset from fingertip, SD | A>N, $p$<0.001 |
| Static finger, dwell proportion to IA, SD | A>N, $p$<0.001 | Whole movement, dwell proportion to IA, SD | A>N, $p$<0.001 |
| Dynamic finger, horizontal offset from fingertip, SD | A>N, $p$<0.001 | Peak acceleration (direct condition), SD | A>N, $p$<0.001 |

**Table S1.** Features selected with Wrapped $t$-test selection method in eye movement and combined datasets. Mean difference column shows whether the mean for a particular feature was greater for autistic (A) or non-autistic (N) class and gives a $p$-value of two-sample $t$-test. SD - standard deviation, diff. — difference.

### Combined dataset

In a dataset in which both kinematic and eye movement features were combined, among most discriminative features nine out of ten were eye movement features (Table S1). All of the nine eye-movement features which were selected from the combined dataset were also selected in the eye movement dataset alone (nine features in the left and right parts of the table match). The single selected kinematic feature was a feature which was also most discriminative when Wrapped $t$-test selection was used on kinematic dataset alone (Fig. 7c in the main text). This observed consistency of selected features in different datasets signifies the stability of the Wrapped $t$-test method.

The single selected kinematic feature measured peak acceleration variability in the direct experimental condition. This is consistent with behavioural results in previous imitation studies which have shown significant between-group movement differences between direct and elevated trials[1–3] and with our finding that the majority of most discriminative features from the kinematic dataset were based on acceleration (see section Discriminative features in the main manuscript). Features selected using Wrapped $t$-test from a combined dataset gave better classification performance than features selected from either

kinematic or eye movement datasets alone. This suggests that the most relevant imitation performance aspects differentiating between autistic and non-autistic individuals were the acceleration variability in direct trials and variability in visual attention to the finger performing movement sequences.

## References


**1.** Gowen, E., Vabalas, A., Casson, A. J. & Poliakoff, E. Instructions to attend to an observed action increases imitation in autistic adults. *Autism* DOI: 10.1177/1362361319882810 (2019).

**2.** Wild, K. S., Poliakoff, E., Jerrison, A. & Gowen, E. Goal-directed and goal-less imitation in autism spectrum disorder. *J. Autism Dev. Disord.* **42**, 1739–1749 (2012).

**3.** Forbes, P. A. G., Pan, X. & Hamilton, A. F. Reduced mimicry to virtual reality avatars in autism spectrum disorder. *J. Autism Dev. Disord.* **46**, 3788–3797 (2016).
